# Supplementary material for: Mediator 1 ablation induces enamel-to-hair lineage conversion in mice through enhancer dynamics
Source: Commun Biol. 2023 Jul 21;6:766. doi: 10.1038/s42003-023-05105-5 (PMC10362024; doi:10.1038/s42003-023-05105-5)
Supplement: Supplementary file 2 — Supplemental Information [file 42003_2023_5105_MOESM2_ESM.pdf]

Supplementary information

Mediator 1 ablation induces enamel-to-hair lineage conversion in mice through enhancer dynamics

Roman Thaler[1,2], Keigo Yoshizaki[3], Thai Nguyen[4], Satoshi Fukumoto[5,6], Pamela Den Besten[7], Daniel D. Bikle[4], Yuko Oda[4]*

[1] Department of Orthopedic Surgery, Mayo Clinic, Rochester, MN, USA
[2] Center for Regenerative Medicine, Mayo Clinic, Rochester, MN, USA
[3] Section of Orthodontics and Dentofacial Orthopedics, Division of Oral Health, Growth and Development, Kyushu University Faculty of Dental Science, Fukuoka, Japan.
[4]Departments of Medicine and Endocrinology, University of California San Francisco and San Francisco Veterans Affairs Health Center, San Francisco, CA, USA
[5] Section of Pediatric Dentistry, Division of Oral Health, Growth and Development, Kyushu University Faculty of Dental Science, Fukuoka, Japan
[6] Division of Pediatric Dentistry, Department of Oral Health and Development Sciences, Tohoku University Graduate School of Dentistry, Sendai, Japan
[7] Department of Dentistry, University of California San Francisco, San Francisco, CA, USA

Table of Content

Supplementary Figures 1-10

Supplemental data 1 for source data behind the graphs shown in Figures 3-7 and supplementary 5 figures 2, 6, 7, 8, 9, 10 shown in 11 separate sheets (separate Excel file).

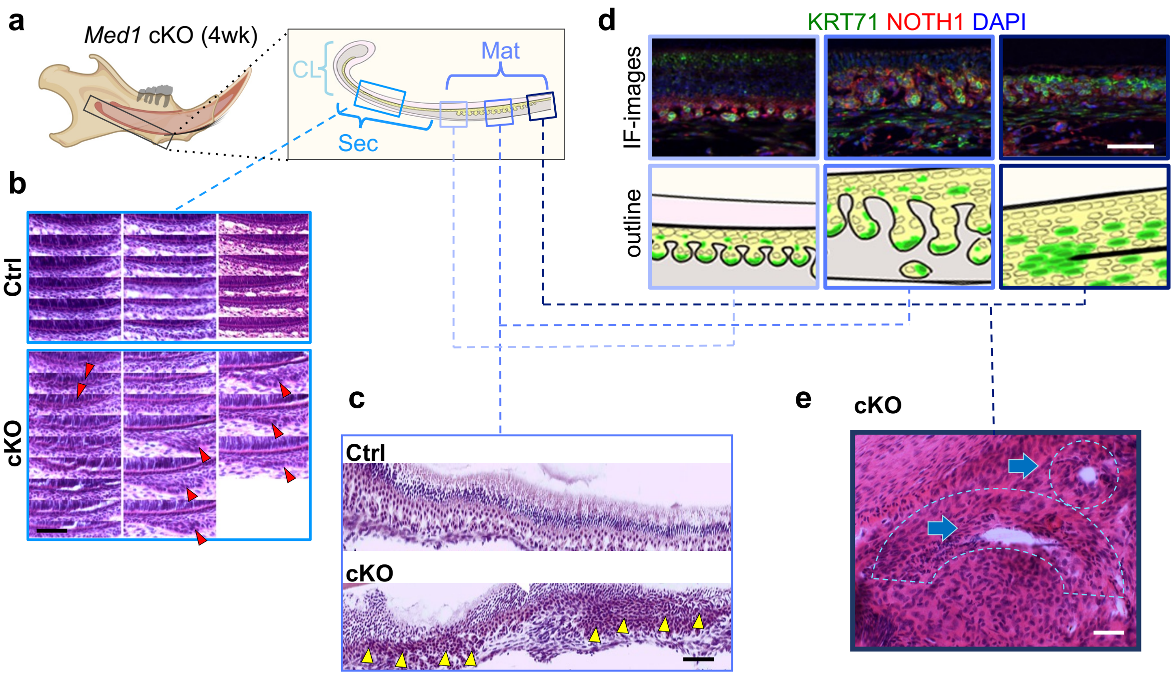

Supplementary Fig. 1: Histological and immuno-histological characterization of *Med1* cKO incisor tissue. a Diagram depicting the differentiation stages of mouse incisors including the location of the cervical loop (CL), the secretory stage (Sec) and the maturation stage (Mat) of dental epithelia. Right, detailed representation of Sec and Mat. **b** Serial sectioning showing progression of abnormal stratum intermedium (SI) development with generation of atypical cell clusters (red triangles) in *Med1* cKO mice. **c** HE staining to show abnormal expansion of papillary layer in cKO (yellow triangles). **d** Top, immuno-fluorescent visualization of KRT71 (green), NOTCH1 (red) and DAPI counterstaining (blue) in SI/stellate reticulum (SR) derived papillary layers at 3 different locations of the Mat stage in *Med1* cKO mice. Bottom, diagram illustrating the locations where the hair marker KRT71 is expressed (green). **e** HE stained sections emphasizing the location of hair generating cell clusters (dotted line) surrounding hair shafts (blue arrows) in *Med1* cKO (4wk). Bar=50 μm and representative images are shown.

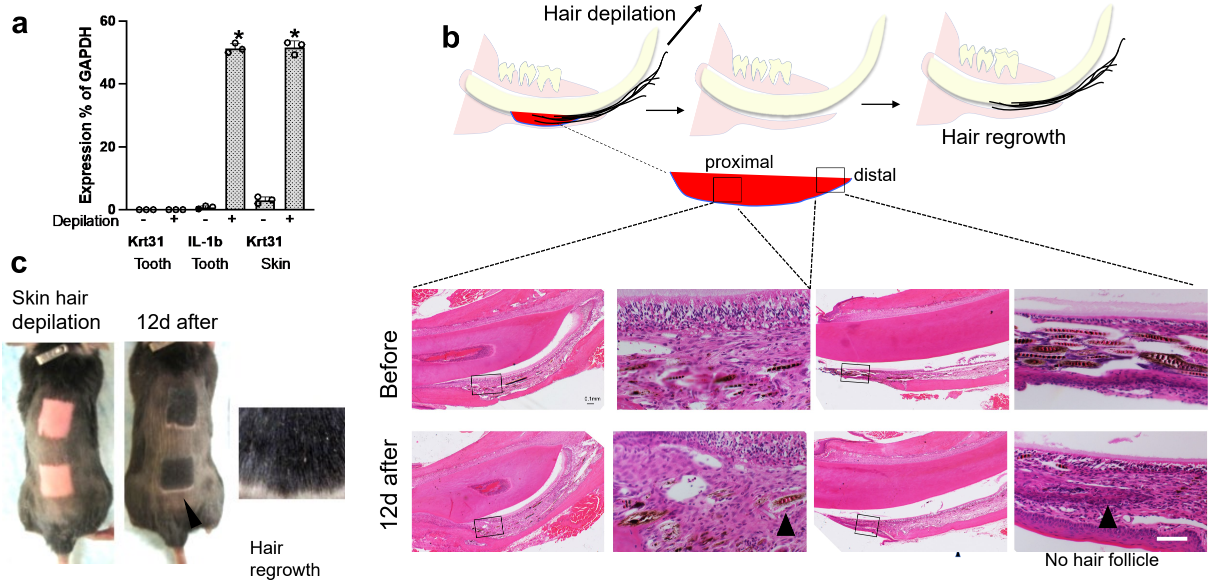

Supplementary Fig. 2: Effects of hair depilation on dental hair regeneration. a Hair marker *Krt31* and *IL-1β* mRNA expression in CL tissues of *Med1* cKO mice (tooth, left) and in normal skin tissue (skin right) before (-) and 8 days after (+) hair depilation. Data shows average and SD of expression relative to GAPDH and the statistical significance (t-test, *p<0.05, *n*=3). **b** Histological assessment of atypical hair generating cell clusters in *Med1* cKO incisors before and 12 days after hair depilation in proximal and distal region of the mandible corresponding to diagram. Boxed area (left) is enlarged (right) to visualize hair generating tissues (black triangles) which do show hair follicle structures even after hair depilation. Bar=50 μm **c** Mouse skin shortly after hair depilation (left) and 12 days post depilation (right), when hair is regrown (arrow) as also shown by the enlarged image.

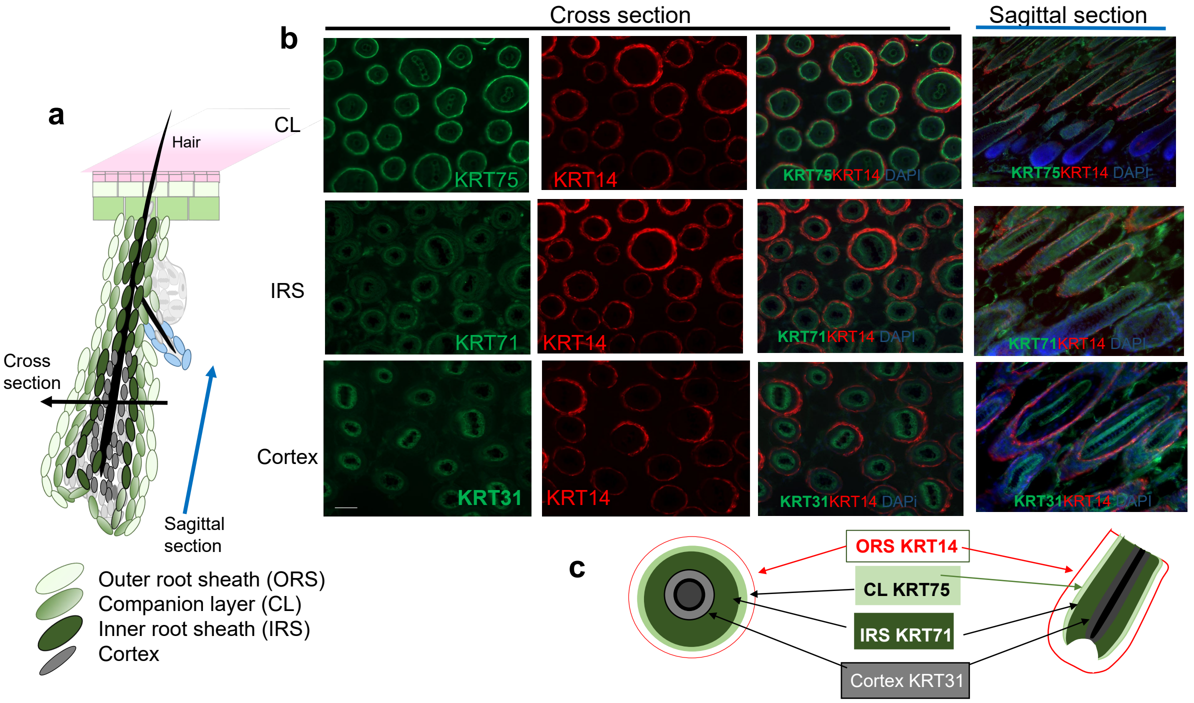

Supplementary Fig. 3: Different hair keratins visualize distinct layers of hair follicles in the skin. a Schematic representation of cross- and sagittal-sections of hair follicles in the skin containing the ORS (light green), the companion layer (Cl, pale green), the IRS (dark green) and the hair cortex (grey) surrounding hair shaft (black). **b** Co-localization of ORS marker Krt14 (red) with other hair keratins including KRT75 (Cl, top panels), KRT71 (IRS, middle panels), and KRT31 (Cortex, lower panels) in cross sections (3 left panels) and with DAPI counter stain (blue) in sagittal sections (far right panel) on cryosections of normal skin (4wk anagen). Bar=50 μm. Representative images are shown, and reproducibility was confirmed in two back skins from two mice. **c** Representation of hair follicle ring structures visualized by different hair keratins at cross sections.

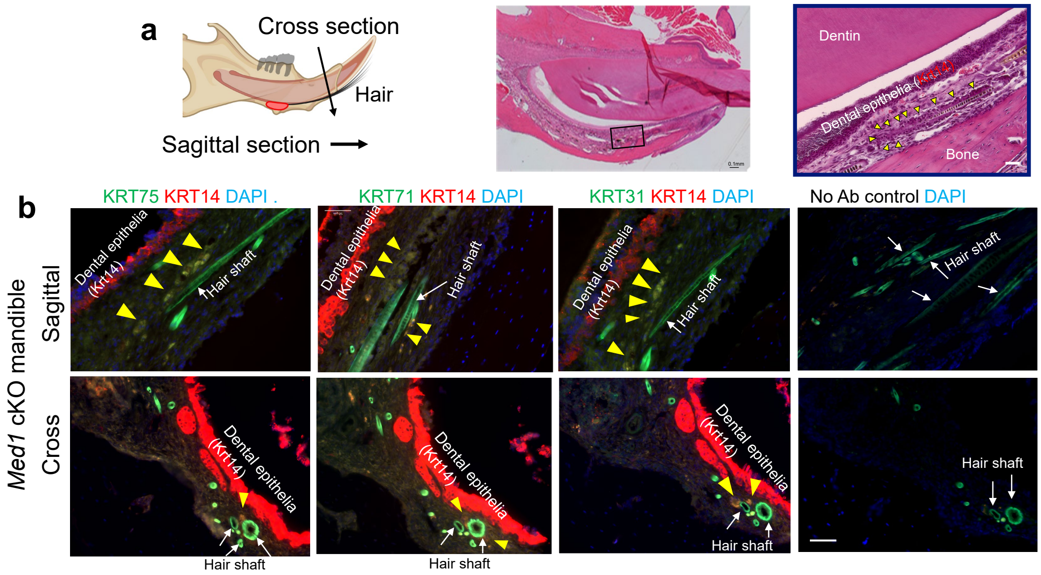

Supplementary Fig. 4: Hair keratin localization on *Med1* cKO incisors. a Schematic representation of cross- and sagittal-sections of hair generating tissues on *Med1* cKO mandibles. Representative HE images of whole mandible (left), and atypical cell cluster area surrounding hair shafts (yellow triangles) (right). **b** Double staining of distinct hair keratins (green), including KRT75 (first panel), KRT71 (second panel) and KRT31 (third panel) with the epithelial marker KRT14 (red) in serial sections. The position of hair shafts is shown by green autofluorescence (seen in no antibody control) below the dental epithelia represented by strong red stain (KRT14), in which we used the condition to over-saturated dental epithelial red signals, so that we can see hair keratin staining overlapped with red KRT14 (yellow) surrounding the hair shafts (yellow triangles). Minimum DAPI blue counter staining is shown to visualize green and red signals. Sagittal (top panels) and cross sections (bottom panels) are shown. No antibody control (far right panels) show autofluorescence of hair shafts. Bar=50μm. Representative images of *Med1* cKO (6 months) are shown and reproducibility was confirmed in two independent anatomical regions in two *Med1* cKO mice.

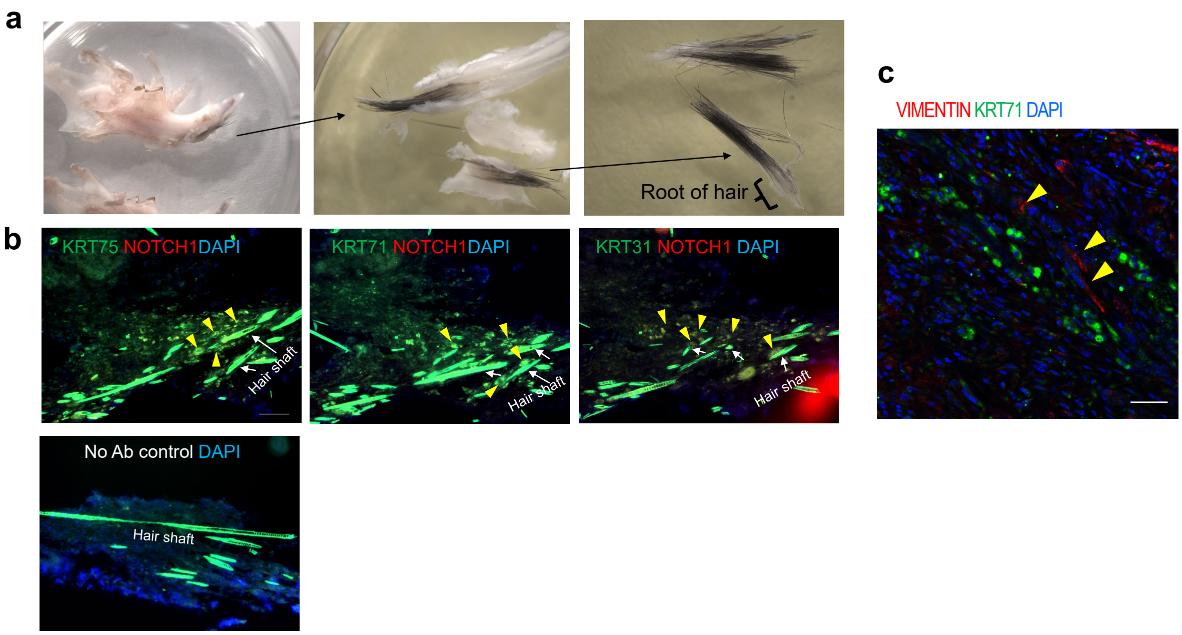

Supplementary Fig. 5: Expression of different hair keratins and the mesenchymal protein VIMENTIN in dissected *Med1* cKO incisor tissue. a Dissection process of hair generating tissues from *Med1* cKO mandible. Picture depicting analyzed root area is shown (bracket, right). **b** Double immuno-staining of different hair keratins KRT75 (left), KRT71 (middle), and KRT31 (right) with dental epithelial SI marker NOTCH1 (red) at serial sections, in which DAPI was minimized to visualize yellow signals (green hair keratin overlapped with red NOTCH1). The DAPI show the presence of tissue in No Ab control (bottom). Hair shafts show strong green autofluorescence, that are intentionally over-saturated so that hair keratin signals are well visualized. The position of hair shafts is shown by white arrows. Scattered hair keratin expression overlapped with NOTCH1 around hair shafts is shown by yellow triangles. **c** Double immuno-staining of VIMENTIN (red, yellow triangle) and hair keratin KRT71 (green) with DAPI. Bar=50 μm. Representative images of *Med1* cKO (6 months) tissues are shown, reproducibility was confirmed using two tissues from 2 *Med1* cKO mice.

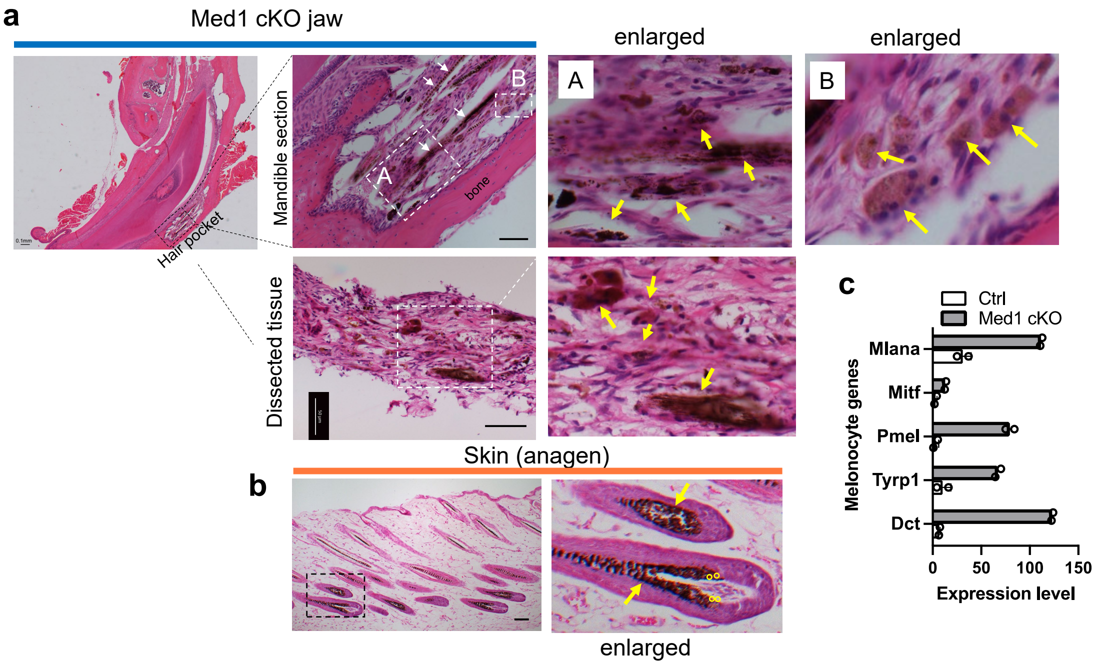

Supplementary Fig 6: Melanin distribution in *Med1* cKO incisor and in normal skin. **a** HE sections of hair generating pocket tissues in *Med1* cKO mandible. Overview picture (left top) and enlarged hair pocket image (top, second panel) in which hair shafts are shown by white arrows. Brown pigmentation is shown by yellow arrows in enlarged A and B sub-sections (top panels). The same brown pigments were identified in dissected tissues from *Med1* cKO mandible (lower panels with enlarged image) as shown in supplementary Fig 5a. **b** Skin section showing melanin accumulation in the hair bulbs (4wk, anagen) and location of bulb melanocytes (yellow circles, enlarged image). Bar = 50 μm, representative images in 2 different pocket areas from 2 *Med1* cKO mice (6 months). **c** mRNA expression of melanocyte marker genes in *Med1* cKO (gray bars) compared to littermate Ctrls (open bars) at CLT (RNA-seq). Expression levels in two independent litters of *Med1* cKO and Ctrl mice (6 CL tissues are pooled in each group) are shown.

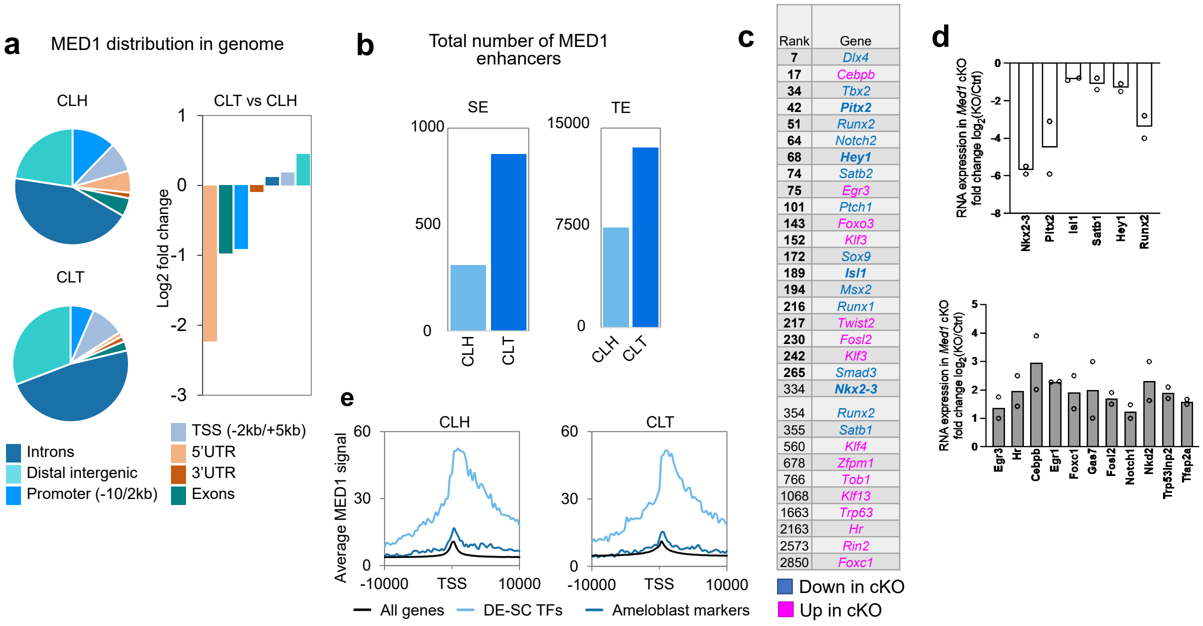

Supplementary Fig. 7: MED1 is differentially distributed between dental stem cells (CLH) and their progenies (CLT) in normal mice. a Left, relative distribution of MED1 binding at different genomic elements in CLH and CLT Ctrl tissues (left); right, shift in MED1 binding at shown elements between CLT and CLH of Ctrl tissues (right) **b** Total number of super enhancers (SE) and typical enhancers (TE) in CLH and CLT Ctrl tissues. The 325 SEs and 871 SEs were identified in the CLH and CLT, respectively. **c** List of transcription factors regulated by MED1 containing enhancers in CLH. Enhancer ranking is noted on the left and colors define regulation of mRNA expression in CLH upon loss of *Med1*. **d** mRNA expression of down-regulated (upper panels) and up-regulated (lower panels) transcription factors in *Med1* cKO CLT was confirmed by using RT-qPCR. Fold changes of mRNA expression in 2 independent litters of *Med1* cKO and Ctrl mice (6 CL tissues were pooled from 3 mice) are shown. **e** Average MED1 occupancy at promoters

(TSS +/- 10kbp) for enamel fate transcription factors (TFs) (pale blue line), ameloblast markers (blue line) and all other genes (black line) in CLH and CLT tissues.

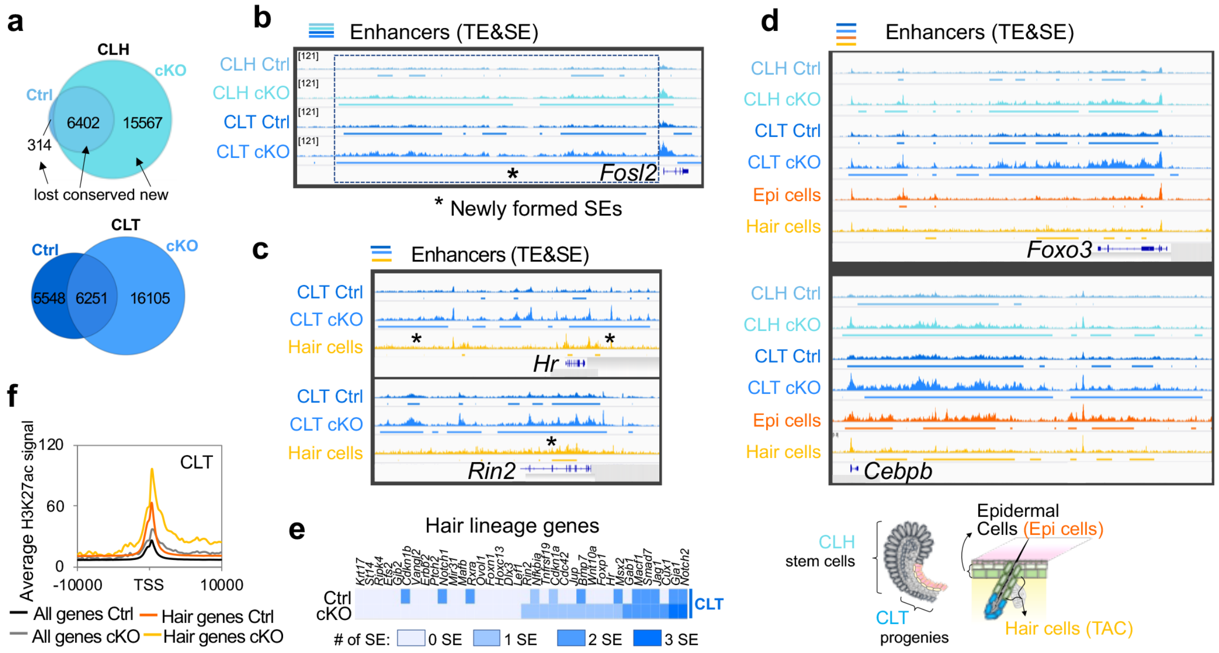

Supplementary Fig. 8: Genomic H3K27ac occupancy at promoters and enhancers in Ctrl and cKO dental tissues. a Numbers of typical enhancers (TEs) in Ctrl and cKO CLH and CLT tissues. Lost, conserved and new TEs in cKO tissues are shown. **b-d** H3K27ac occupancy around the loci of epidermal *Fosl2* (**b**), hair lineage driving *Hr* and *Rin2* (**c**), and epidermal transcription factors *Foxo3* and *Cebpb* (**d**) in Ctrl and *Med1* cKO CLH and CLT tissues. Newly formed super-enhancers (SEs) in cKO are marked with asterisks (*). Enhancer profiles are compared with enhancer profiles (H3K27ac) from skin derived epidermal cells (Epi cells) and hair follicle transient amplifying (TAC) cells (Hair cells). Schematic representation of the anatomical locations of dental and skin epithelial is included. **b-d**, genome annotations are shown in Supplementary Data 1 (Excel). **e** Number of H3K27ac super-enhancers around genomic loci for hair lineage genes after

Med1 loss in CLT tissues. **f** Average H3K27ac occupancy at promoters (TSS +/- 10kbp) for hair lineage genes in CLT tissues in *Med1* cKO and Ctrl mice.

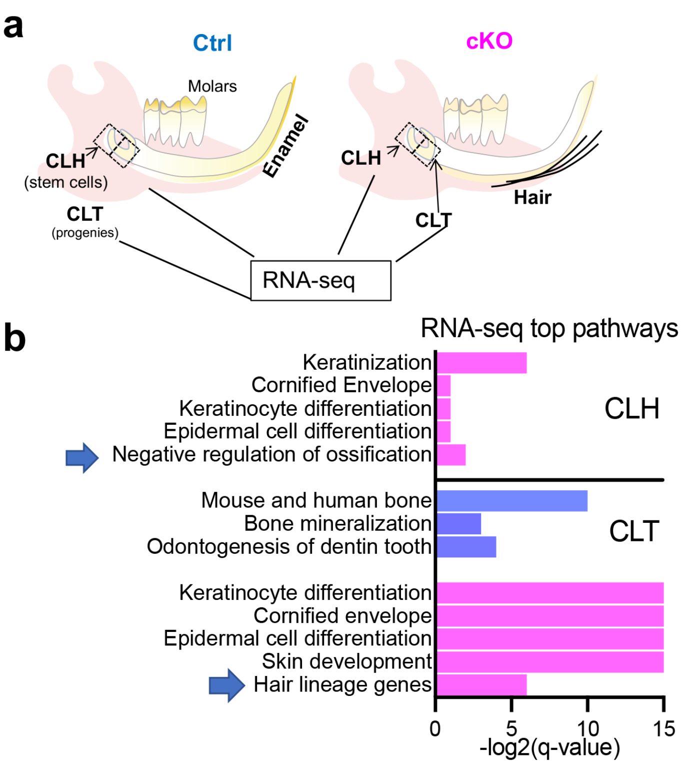

Supplementary Fig. 9: Functional annotation for differentially expressed genes in *Med1* cKO versus Ctrl dental stem cells (CLH) and their progenies (CLT). **a** Top, schematic diagram illustrating the collected tissues for the RNA-Seq experiment using two litters of 4 weeks-old *Med1* cKO and Ctrl mice. **b** GO-based annotations representing up-regulated gene sets are shown in pink, down-regulated gene sets are shown in blue. Blue arrows show relevant pathways for hair formation (bottom) and enamel dysplasia (top) observed in *Med1* cKO mice. The values for keratinocyte differentiation, cornified envelope, epidermal cell differentiation and skin development are over 15 (Supplemental Data 1).

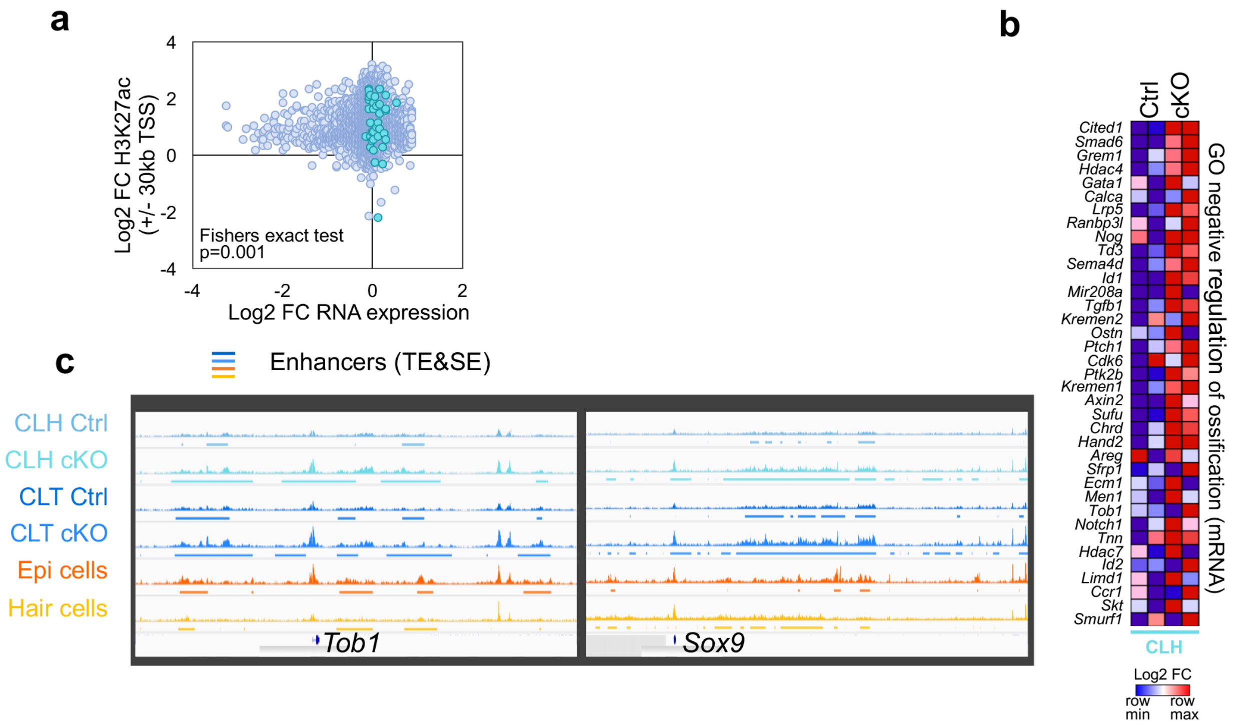

Supplementary Fig. 10: Loss of *Med1* perturbs ossification pathways in dental tissues. **a** Correlation between gene expression and H3K27ac promoter occupancy (TSS +/-30 kb) in *Med1* cKO vs Ctrl CLT tissues. Blue dots highlight the gene-set 'negative regulation of ossification', pale blue dots represent all other genes. **b** Heatmap showing the changes in RNA expression for the gene set 'Go negative regulation of ossification' in Ctrl and *Med1* cKO CLH tissues. **c** Chip-seq enhancer profiles (H3K27ac) around loci of ossification inhibitors *Tob1 and Sox9* (mm10). Here, enhancers (colored underlines) expand in size or height after loss of *Med1* in CLH and CLT tissues. As a comparison, H3K27ac profiles for skin derived epidermal cells (Epi cells) and hair follicle TAC cells (Hair cells) are shown.
