# Supplementary material for: Mediator 1 ablation induces enamel-to-hair lineage conversion in mice through enhancer dynamics
Source: Commun Biol. 2023 Jul 21;6:766. doi: 10.1038/s42003-023-05105-5 (PMC10362024; doi:10.1038/s42003-023-05105-5)
Supplement: Supplementary file 3 — Description of Additional Supplementary Files [file 42003_2023_5105_MOESM3_ESM.pdf]

Description of Additional Supplementary Files

File name: Supplementary Data 1

Description: The source data behind the graphs shown in Figures 3-7 and supplementary figures 2, 6, 7, 8, 9, 10 shown in 11 separate sheets.
