# Supplementary material for: Mediator 1 ablation induces enamel-to-hair lineage conversion in mice through enhancer dynamics
Source: Commun Biol. 2023 Jul 21;6:766. doi: 10.1038/s42003-023-05105-5 (PMC10362024; doi:10.1038/s42003-023-05105-5)
Supplement: Supplementary file 5 — Reporting Summary [file 42003_2023_5105_MOESM5_ESM.pdf]

nature portfolio

Corresponding author(s): Yuko Oda

Last updated by author(s): 2023 June, 19

Reporting Summary

Nature Portfolio wishes to improve the reproducibility of the work that we publish. This form provides structure for consistency and transparency in reporting. For further information on Nature Portfolio policies, see our Editorial Policies and the Editorial Policy Checklist.

Statistics

For all statistical analyses, confirm that the following items are present in the figure legend, table legend, main text, or Methods section.

n/a	Confirmed	
☐	☒	The exact sample size (*n*) for each experimental group/condition, given as a discrete number and unit of measurement
☐	☒	A statement on whether measurements were taken from distinct samples or whether the same sample was measured repeatedly
☐	☒	The statistical test(s) used AND whether they are one- or two-sided<br>*Only common tests should be described solely by name; describe more complex techniques in the Methods section.*
☐	☒	A description of all covariates tested
☐	☒	A description of any assumptions or corrections, such as tests of normality and adjustment for multiple comparisons
☐	☒	A full description of the statistical parameters including central tendency (e.g. means) or other basic estimates (e.g. regression coefficient) AND variation (e.g. standard deviation) or associated estimates of uncertainty (e.g. confidence intervals)
☐	☒	For null hypothesis testing, the test statistic (e.g. *F*, *t*, *r*) with confidence intervals, effect sizes, degrees of freedom and *P* value noted<br>*Give P values as exact values whenever suitable.*
☒	☐	For Bayesian analysis, information on the choice of priors and Markov chain Monte Carlo settings
☒	☐	For hierarchical and complex designs, identification of the appropriate level for tests and full reporting of outcomes
☒	☐	Estimates of effect sizes (e.g. Cohen's *d*, Pearson's *r*), indicating how they were calculated

Our web collection on statistics for biologists contains articles on many of the points above.

Software and code

Policy information about availability of computer code

Data collection	Applied Biosystems 7500 Real-Time PCR System and QuantStudio Real-time PCR system (Thermofisher) were used for real time PCR for RT-PCR and Chip-seq PCR experiments.<br>LSM 510 inverted confocal microscope or AXIO fluorescent microscope (Carl Zeiss) was used for imaging.<br>DNA sequencing for Chip-seq was conducted by Illumina Hiseq 4000 instrument in the UCSF Center of Advanced Technology core (CAT) facility<br>Microarray was conducted in the UCLA core facility using Illumina microchip (Mouse Ref-8 v2.0).<br>RNA-Seq was performed on an Illumina Hiseq 4000 in the Mayo Clinic genome facility sequencing core.
Data analysis	QPCR data were collected using the Applied Biosystem's softwares installed in the instrument, analyzed by Excel (Microsoft) using t-test, and visualized by Graphpad Prism version 8 or 9. Bright-field microscopic images were analyzed using ImageJ software (NIH) and performed quantitative analysis by using bioquant system in Bone core facility in VA Medical Center San Francisco. Microarray gene expression profiles were analyzed using a lllumina beads chip based gene array (Mouse Ref-8 v2.0 Ambion). Sample preparation, labeling and array hybridizations were conducted according to standard protocols by the UCLA Neuroscience Genomics Core Facilities. The data were normalized in the Genome Studio (Illumina). The fold changes (log) in gene expression of Med1 KO over control were calculated (average intensity, p-values, and standard deviation). The data were then analyzed by using the IPA software (Ingenuity) via data transformation by setting the score and the p-value less than 0.005. Several analyses performed to predict upstream regulators and affected pathways were conducted using IPA software. The upstream molecules were listed, which are potentially responsible for the observed changes in gene expression. The statistical significance of potential upstream regulators was evaluated by the z-score and the p-values calculated through the algorithm installed in IPA software. Heat maps were prepared by using MeV software using fold changes (KO/CON).<br>Raw reads from ChIP-seq experiments were assessed for run quality using fastqc version 0.72. Mapping of ChIP-seq data to reference genome was performed using BWA Galaxy version 0.7.15.1. Peak calling was performed with MACS2 callpeak Galaxy version 2.1.0.20140616.0 and differential peak analysis with DiffBind Galaxy version 2.6.6.4 or 2.10.0. Peak size and location was visualized with IGV version 2.3.92.MAYO.

Genomic ChIP-Seq related peak distribution was assessed with CEAS software (Version1.0.0, Cistrome, Liu Lab). Super-enhancer assessment was performed by using the package the software NaviSE (Ascension M e al., BMC Bioinformatics 2017). Overlapping SE between samples and/or treatments were assessed with Bedtools (version 2.29). ChIP-Seq based gene-ontology was performed using GREAT version 3.0.0 (Bejerano Lab, Stanford; http://great.stanford.edu/public/html/). Hierarchical clustering and heat map generation was executed with Morpheus (Broad Institute, https://software.broadinstitute.org/morpheus/). Statistical data analysis and figure generation was performed using GraphPad Prism software, version 9.4.1 as well as Microsoft excel.

For manuscripts utilizing custom algorithms or software that are central to the research but not yet described in published literature, software must be made available to editors and reviewers. We strongly encourage code deposition in a community repository (e.g. GitHub). See the Nature Portfolio guidelines for submitting code & software for further information.

Data

Policy information about availability of data

All manuscripts must include a data availability statement. This statement should provide the following information, where applicable:

- Accession codes, unique identifiers, or web links for publicly available datasets
- A description of any restrictions on data availability
- For clinical datasets or third party data, please ensure that the statement adheres to our policy

The array data were submitted to a public database (GEO/NCBI/NIH http://www.ncbi.nlm.nih.gov/geo). Data for Med1 cKO (4wk), are available with accession numbers GSE50503 under the super-series GSE50504. The Chip-seq data were deposited with accession number GSE221565 and RNA-seq data with GSE232190.

Human research participants

Policy information about studies involving human research participants and Sex and Gender in Research.

Reporting on sex and gender	No human research
Population characteristics	NA
Recruitment	NA
Ethics oversight	NA

Note that full information on the approval of the study protocol must also be provided in the manuscript.

Field-specific reporting

Please select the one below that is the best fit for your research. If you are not sure, read the appropriate sections before making your selection.

☒ Life sciences ☐ Behavioural & social sciences ☐ Ecological, evolutionary & environmental sciences

For a reference copy of the document with all sections, see nature.com/documents/nr-reporting-summary-flat.pdf

Life sciences study design

All studies must disclose on these points even when the disclosure is negative.

Sample size	Histology experiments using Med1 cKO and littermate control mice were repeated with at least two litters, and reproducibility was confirmed. The experiments using cultured dental epithelia were conducted in duplicates for microarray, and reproducibility was confirmed. Histological analyses were performed using 2 sections per mouse per each group of cKO and Ctrl, and representative images are shown. Statistical significance was calculated using software integrated methods or two-tailed unpaired Student's t-test. If not differently noted, differences with a p-value of less than 0.05 were considered as statistically significant.
Data exclusions	No data exclusion
Replication	Chip-seq experiments were conducted in duplicate, in which cervical loop tissues from 2-4 mice (Med1 cKO and littermate control) are pooled for one Chip experiment (total 4-8 cervical loop tissues) and repeated the same experiments using a different litter. Average profiles are shown in IGV genome browser. Super-enhancer analysis was conducted on averaged peaks using NaviSE, and binding motif analysis was conducted by Homer analysis with known motifs.
Randomization	Mice were randomly allocated in the treatment group after determining genotypes and gender. For microscopy image capturing and analysis, selected areas were chosen randomly. For other experiments, randomization was not applicable and/or relevant as these experiments did not involve clinical trials or population based studies. However, for the in vitro performed experiments, cells were cultured under the described conditions and unbiasedly seeded to well positions and treatments. In addition, cell harvesting, processing and analysis was performed in random order.

Blinding	Tissue and cell culture collections for molecular analysis were not performed blind as subsequent experiments were carried out by the same

Reporting for specific materials, systems and methods

We require information from authors about some types of materials, experimental systems and methods used in many studies. Here, indicate whether each material, system or method listed is relevant to your study. If you are not sure if a list item applies to your research, read the appropriate section before selecting a response.

Materials & experimental systems

n/a	Involved in the study
☐	☒ Antibodies
☒	☐ Eukaryotic cell lines
☒	☐ Palaeontology and archaeology
☐	☒ Animals and other organisms
☒	☐ Clinical data
☒	☐ Dual use research of concern

Methods

n/a	Involved in the study
☐	☒ ChIP-seq
☒	☐ Flow cytometry
☒	☐ MRI-based neuroimaging

Antibodies

Antibodies used	H3K27ac (Abcam ab 4729, Lot GR3211959-1, GR3303561-2) 3ug/Chip IP, Med1 (Bethyl Laboratories, A300-793A Lot 9, 10) 4.5 ug/ Chip IP, Loricrin (Biolegend former Covance 905104,PRBP-145P Lot 14892401)(1:200), K14 (Krt14 Biolegend former Covance 905304 PRB-155P Lot 14834401)(1:1000), K71 (Krt71) (Progen GP-K6irs1 Lot 208291),(1:200) Notch1 (Cell signaling 3447)(1:200), KRT75 (ARP, anti-cytokeratin K6HF (K75) Lot 20411-01, 1:100), or KRT31 (Progen, Lot 203211, 1:100).
Validation	All the antibodies are verified by the vendors. We tested Lor, K14 (Krt14), K71 (Krt71), K75 (Krt75), Krt31 and Notch1 for immuno-staining by using our samples if antibodies provide us reasonable signals by sited dilutions. We also tested Med1 and H3K27ac antibodies in Chip-seq by preliminary Chip-qPCR using known primers to verify if these antibodies efficiently immuno-precipitate the appropriate genomic sites by comparing negative control samples (Input). We also verify them by comparing signal levels using negative control PCR primers that are provided by Chip-seq kit (Diagenode).

Animals and other research organisms

Policy information about studies involving animals; ARRIVE guidelines recommended for reporting animal research, and Sex and Gender in Research

Laboratory animals	Conditional Med1 knockout (cKO) mice were generated by mating floxed (exon 8-10) Med1 mice (C57/BL6 background) with keratin 14 (Krt14) promoter driven Cre recombinase mice (The Jackson Laboratory, C57/BL6 background). Genotyping was determined by PCR and Cre negative littermate mice were served as controls (Ctrl). All mice were housed in a selected pathogen-free barrier environment with ad libitum access to food and water, 12-hour light and dark cycles, temperature between 20-26C.
Wild animals	The study did not involve wild type animals.
Reporting on sex	Findings reported in this study apply to both sexes as study design and experimental setups included both, male and female animals.
Field-collected samples	Our study did not include field collected samples.
Ethics oversight	We collected mouse specimen under protocol number 20-019 (Daniel D. Bikle) that were approved by the Institutional Animal Care and Ethics Committee at the San Francisco Department of Veterans Affairs Medical Center. Our staffs conduct our experiments and received annual training for ethics conducts under the ethics, animal welfare and IAUCU oversight. We collected samples at end point and no surgery procedure is included (no category C experiments).

Note that full information on the approval of the study protocol must also be provided in the manuscript.

ChIP-seq

Data deposition

☒ Confirm that both raw and final processed data have been deposited in a public database such as GEO.

☒ Confirm that you have deposited or provided access to graph files (e.g. BED files) for the called peaks.

Data access links<br>*May remain private before publication.*	The ChIP-seq data were deposited and available with accession number GSE221565 at the following URL: https:// www.ncbi.nlm.nih.gov/geo/query/acc.cgi?acc=GSE221565).
Files in database submission	Chip-seq GSE221565

Files in database submission

GSM6886371 Ctrl CLH Med1
GSM6886372 Ctrl CLT Med1
GSM6886373 Ctrl CLH H3K27ac
GSM6886374 Ctrl CLT H3K27ac
GSM6886375 Med1 cKO CLH H3K27ac
GSM6886376 Med1 cKO CLT H3K27ac
GSM6886377 Input CL
GSM6886378 WT keratinocyte H3K27ac
GSM6886379 Input_keratinocyte
All the data will be available after June 22, 2023.

Genome browser session
(e.g. UCSC)

N/A. We used IGV genome browser.

Methodology

Replicates

Average of duplicate data that are derived from two independent litter of Med1 KO and Ctrl (2-4 mice, pooled 4-6 CL tissues each).

Sequencing depth

Uniquely mapped sequences per total was over 85% for H3 and over 75% for Med1
Ctrl CLH Med1 rep1, total reads 12132984, uniquely mapped reads 10843553, length: 50 bp, single end

Ctrl CLH Med1 rep2, total reads 30349324, uniquely mapped reads 26305412, length: 50 bp, single end

Ctrl CLT Med1 rep1, total reads 11614010, uniquely mapped reads 10627101, length: 50 bp, single end

Ctrl CLT Med1 rep2, total reads 18042268, uniquely mapped reads 16557063, length: 50 bp, single end

Ctrl CLH H3K27ac rep1, total reads 42943319, uniquely mapped reads 38857028, length: 50 bp, single end

Ctrl CLH H3K27ac rep2, total reads 81309078, uniquely mapped reads 75175653, length: 50 bp, single end

Ctrl CLT H3K27ac rep1, total reads 28479608, uniquely mapped reads 25701187, length: 50 bp, single end

Ctrl CLT H3K27ac rep2, total reads 76306333, uniquely mapped reads 71366872, length: 50 bp, single end

Med1 cKO CLH H3K27ac rep1, total reads 72255459, uniquely mapped reads 65671005, length: 50 bp, single end

Med1 cKO CLH H3K27ac rep2, total reads 88441873, uniquely mapped reads 83732204, length: 50 bp, single end

Med1 cKO CLT H3K27ac rep1, total reads 51173258, uniquely mapped reads 47931133, length: 50 bp, single end

Med1 cKO CLT H3K27ac rep2, total reads 42239040, uniquely mapped reads 40214201, length: 50 bp, single end

Input 1, total reads 42297924, uniquely mapped reads 31890624, length: 50 bp, single end

Input 2, total reads 19515039, uniquely mapped reads 15981156, length: 50 bp, single end

Input 3, total reads 51124264, uniquely mapped reads 42035754, length: 50 bp, single end

Input 4, total reads 45093006, uniquely mapped reads 37415992, length: 50 bp, single end

Antibodies

H3K27ac (Abcam ab 4729, Lot GR3211959-1, GR3303561-2) 3ug/Chip IP,
Med1 (Bethyl Laboratories, A300-793A Lot 9, 10) 4.5 ug/Chip IP

Peak calling parameters

Peaks were defined using MACS2 callpeak Galaxy version 2.1.0.20140616.0 with parameters -g mm and -p 1e-05

Data quality

evaluated by peak at 5% FDR, peaks with fold enrichment >5, detailed data will be provided.

Software

Fastq files from ChIP-Seq experiments were assessed for run quality using fastqc version 0.72. Mapping to the reference genome mm10 was performed using BWA Galaxy version 0.7.15.1 using standard settings. Peak calling was performed with MACS2 callpeak Galaxy version 2.1.0.20140616.0 using -g mm and -p 1e-05 and differential peaks analysis with DiffBind Galaxy version 2.6.6.4 or 2.10.0. Genomic ChIP-Seq related peak distribution was assessed with CEAS software (Version 1.0.0, Cistrome, Liu Lab). Super-enhancer assessment was performed using the package ROSE as integrated in the NaviSE software package (Ascension M et al., BMC Bioinformatics 2017) by stitching together neighboring peaks within 12.5 kb from each other while excluding regions +/- 2000 bps from any transcription start site. Overlapping SE between samples were assessed by Bedtools (version 2.29). ChIP-Seq based gene-ontology was performed using GREAT version 3.0.0 (Bejerano Lab, Stanford; http://great.stanford.edu/public/html/).
